# Intra-operative 5-FU in Glaucoma Surgery: A Nigerian Teaching Hospital Experience

**DOI:** 10.4103/0974-9233.51993

**Published:** 2008

**Authors:** Bernice O Adegbehingbe, H.Onakpoya Oluwatoyin

**Affiliations:** From the Ophthalmology Unit, Department of Surgery, College of Health Sciences, Obafemi Awolowo University; Ile-Ife, Osun State, Nigeria

**Keywords:** trabeculectomy, 5-flouracil, anti-metabolites, effect and complications

## Abstract

**Background:**

5-Fluorouracil (5-FU) is an anti-metabolite used as an adjunct during the initial stages of a trabeculectomy to prevent excessive post-operative scarring and thus reduce the risk of failure.

**Objectives:**

To assess the effects of intra-operative 5-FU in trabeculectomy in Nigerians.

**Methods:**

Trabeculectomy with an intra-operative sponge soaked with 5-FU was performed in 49 eyes of 34 patients. The reduction in intra-ocular pressure (IOP), Visual acuity changes and complications were noted.

**Results:**

The mean presenting and preoperative IOP was 35.6+/-12.5mmHg and 25.5+/-6.6mmHg respectively. The mean post-operative IOP was 10.0+/- 5.2mmHg on the second post-operative day and 16.5+/7.3mmHg at 3 months. Post-operative complications included ocular hypotony in 4 (25 percent), shallowing of anterior chamber in 3 (18.8 percent), hyphema and cystic bleb in 2 (12.5 percent) each. None had bleb failure. 5 FU appears to reduce the relative risk of failure of trabeculectomy in those undergoing surgery for the first time (relative risk 0.29, 95 percent confidence interval 0.16 to 0.53).

**Conclusions:**

The use of 5-FU during trabeculectomy is associated with significant benefit in terms of intraocular pressure lowering beyond 3 months postoperatively. The results of surgery in Nigerian patients compare well with other reported series.

Intra-ocular pressure (IOP) is one of the only risk factor for glaucoma that can be manipulated to alter the cause of the disease. While great success has been achieved with medical treatment; surgery still remains the mainstay of treatment for glaucoma especially among black African patients due to the unavailability and high cost of topical therapy.

Trabeculectomy is performed as a treatment for glaucoma to lower the intra-ocular pressure. However, the procedure tend to fail over time because of fibroblastic proliferation and subconjunctival fibrosis that occur during normal healing.^1^

In order to reduce failure rates adjunctive use of antifibrotic agents with glaucoma surgery significantly reduces the risk of bleb failure, although their use has been associated with a number of complications.^2^–^5^ 5-Fluorouracil (5-FU) is an anti-metabolite used during the initial stages of a trabeculectomy to prevent excessive post-operative scarring and thus reduces the risk of failure. Many other modalities of preventing bleb failure are now being used in the more developed countries, however, the use of 5-FU still remains the only readily available, affordable and easily accessible option in Nigeria.

This study reports the effect of intra-operative 5-FU application during primary trabeculectomy on IOP in this African population.

## Materials and Methods

This was a prospective study of all patients with glaucoma who had trabeculectomy with intra-operative use of 5-FU at the Obafemi Awolowo University Teaching Hospital, Ile-Ife, Nigeria between January 2002 and December 2004.

Approval for the study was obtained from the Research and Ethics committee of the Obafemi Awolowo University Teaching Hospital Complex, Osun State, Nigeria. All participants gave informed consent.

All patients were examined pre-operatively for IOP (Applanation), visual acuity (Snellen's), gonioscopy, and visual field (Humphreys). Patients were initially placed on medical therapy for established glaucoma and immediately advised to have surgery as soon as possible. Post-operatively, patients were evaluated on day 2, the first week and in the first month for anterior chamber formation, IOP measurement and Visual acuity.

Thereafter they were followed up every three months for I year and they are still currently being followed by measuring IOP, visual acuity, visual field every 6 months till date. Additional anti-glaucoma medications were administered in those with poor control of IOP. Success was determined by post operative IOP 20 mmHg or less without anti-glaucoma medication at the time of examination.

The technique of trabeculectomy used in all patients was Watson's^6^ modification of Cairn's procedure combined with the Luntz modification.^7^ A fornix based conjunctival flap was raised followed by tenectomy. A rectangular limbal based superficial scleral flap incision (5× 4mm) was dissected until the surgical limbus was seen.. The conjunctival and the superficial scleral flap were then pulled over 4mm×3mm piece of weckcel sponge saturated with 5-FU (50mg/ml) for a total of 5minutes.^8^ After the sponge was removed, the area was irrigated copiously with 5 ml of normal saline. The trabeculectomy was then completed with a rectangular deep corneo-scleral block excision (3×2mm). A peripheral iridectomy was made before closing the superficial scleral flap with 8/0 virgin silk; the conjunctiva was sutured with 8/0 virgin silk. Subconjunctival gentamycin (20mg) and dexamethasone 10mg were injected into the inferior fornix, followed by topical chloramphenicol and atropine 1 percent drops before the eye was padded. Post operative antibiotics (Chloramphenicol), mydratics (cyclopentolate), and steroid drops were used. Three consultant ophthalmologists performed the surgeries.

## Results

Trabeculectomy, using an intra-operative sponge soaked in 5 percent 5-FU was performed on 49 eyes of 34 patients. There were 20 males and 14 females with a M: F ratio of 1.4:1. The age range was 8 to 78years; mean age was 39.6 +/- 14.5 years (SD).

Most of the eyes 44 (89.8 percent) had chronic open angle glaucoma while 5 (10.2 percent) had chronic narrow angle glaucoma. Most of the eyes 44 (89.8 percent) had 5-FU-augmented trabeculectomy at the first surgery while 5 (10.2 percent) eyes had 5-FU application during a repeat surgery.

The presenting intra-ocular pressure ranged from 18 to 56 mmHg, with a mean of 35.6+/- 12.5mmHg (SD). The mean post-operative IOP was 10.0+/- 5.2mmHg on the second post-operative day and 16.5+/7.3mmHg at 3 months. The mean post-operative IOP was 17.4+/- 8.5mmHg and 18.5+/- 6.8mmHg at 6 months and 12 months respectively. Intraocular pressure significantly dropped from mean =25.5+/-6.6mmHg before surgery to mean=18.5+/- 6.8mmHg (p <0.0001)12 months after surgery.

Table 1 shows that on the 2^nd^ post-operative day, 45 (91.8 percent) eyes had a post-operative IOP of 20mmHg or less while 4 (8.9 percent) eyes had IOP of over 21mmHg. At 3 months post-operative visit IOP ranged from 6 to 20 in 41 (83.7 percent) eyes. Postoperative IOP was less than 20mmHg in 40 (81.6 percent) eyes and 41 (83.7 percent) eyes at 6 months and 12 months respectively. Table 2 shows the comparison of preoperative and post-operative visual acuity. Visual acuity improved post-operatively in 5 (10.2 percent) eyes, worsened in 5(10.2 percent) eyes and remained the same in the rest.

**Table 1:** Comparison of Pre-operative and Post-operative IOP in 49 Eyes

IOP (mmHg)	Number of Eyes								
	Presenting	Pre-Op			Post-Operative			
			2 days	2 weeks	4 weeks	3 months	6 months	12 months
10	-	-	18	12	10	10	6	5

11-20	3	10	27	31	29	31	34	36

21-30	15	31	4	6	10	4	3

31-40	20	8	-	-	-	2	5	5

41-50	9	-	-	-	-	-	-	-

> 50	2	-	-	-	-	-	-	-

Success Rate(%)	-	20.4	91.8	87.8	79.6	83.7	81.6	83.7

**Table 2:** Comparison of Pre-operative and Post-operative VA

Visual Acuity	Number of Eyes(%)	
	Pre-Operative	Post-Operative
< 6/9	14(28.6)	18(36.7)

6/12-6/18	19(39.7)	15(30.6)

<6/18-6/60	11(22.5)	12(24.5)


<6/60-3/60	3(6.1)	3(6.1)

<3/60-PL	2(4.1)	1(2.0)

Total	49(100)	49(100)

Post-operative complications included ocular hypotony (25.0 percent), shallowing of anterior chamber (18.8 percent), Hyphema (12.5 percent), cystic bleb (12.5 percent) (Table 3). 5-FU appeared to reduce the relative risk of failure of trabeculectomy in those undergoing surgery for the first time (relative risk 0.26, 95 percent confidence interval 0.13 to 0.43). Mean intra-ocular pressure was significantly reduced at 12 months in all those who received 5-FU.

**Table 3:** Complications of Trabeculectomy with 5-FU (n = 16)

Complications	Number of Eyes(%)
Hypotony	4(25.0)

Shallow anterior chamber	3(18.8)

Increased IOP	2(12.5)

Hyphema	2(12.5)

Cataract	2(12.5)

Cystic Bleb	2(12.5)

Ptosis	1(6.3)

## Discussion

The effectiveness of glaucoma surgery is closely related to the degree of scarring. Bleb failure often occurs over time because of fibroblastic proliferation and subconjunctival fibrosis which occurs during normal process of wound healing. Adjunctive use of anti-fibrotic agents such as 5-FU or Mitomycin C at the sight of surgery significantly reduces the risk of bleb failure.^9^,^10^

Topical application of 5-FU was preferred in this study because of it's advantages over post-operative injection of the same. The former is associated with less frequent patients' visits, no risk of ocular perforation nor corneal epithelia abnormalities and decreased discomfort to the patients.^11^,^12^

The use of 50mg/ml concentration of 5-FU for sponge dabbing the sclera surface was due to the tested efficacy of this dosage in post-operative application in the blacks.^12^,^13^

In our study mean intra-ocular pressure was significantly reduced at 12 months. The success rates recorded were 83.7 percent and 81.6 percent at 3 months and 6 months respectively. These findings are in agreement with the report of other workers. Bayeroju and Ubah recorded a success rate of 85,7 percent at six months^1^4 while Lanigan et al recorded a success rate of 91 percent with limited follow-up (3-9months).^15^ In the first year follow-up of the Fluorouracil filtering surgery study group (FFSS) there was a 73 percent success rate^16^ while Singh et al^13^ and Egbert et al^12^ reported a success rate of 73 percent and 83 percent respectively in the black population studies. The short term success rate in terms of IOP control using 5-FU is quite good. Previous workers reported that long term follow-up suggests that after 2 years the success rate falls, presumably due to slowly replicating fibroblasts.^16^

In our study, the longest follow up period of 2 years was recorded for only a few of the patients. Follow-up visit in our eye clinic is very poor. Most of the patients stop coming to hospital once they have a slight relieve in their symptoms.

Visual acuity improved post-operatively in 5 eyes (3 eyes improved by 1 line, while 1 eye improved from 3/60 to 6/12 and another from 2/60 to 6/60), worsened in 5 eyes (2 due to advancing cataract) and remained the same in the rest. Visual acuity fell by 2-3 lines in those who had advancing cataracts.

Early postoperative complications which were mainly transient (shallow anterior chamber, hypotony, Hyphema and increased IOP) occurred in 11 eyes (22.5 percent) and this was associated with postoperative loss of 2 or more lines of Snellen acuity (OR = 1.08, 95 percent CI 1.13-1.32, P= 0.02). Other complications include progressing cataract formation and ptosis. No significant permanent sight threatening complications was detected but it is likely the sample size was not large enough or of sufficient duration to address the long term risk of bleb infection and endophthalmitis, which has been reported in observational studies. Late complications recorded in other studies were endophthalmitis and late bleb breakdown.^15^ None of the eyes in our series developed either of these complications.

## Conclusion

The use of 5-FU during trabeculectomy is associated with significant benefit in terms of intraocular pressure lowering beyond 3 months postoperatively. The results of surgery in Nigerian patients compare well with other reported series.
